# Copy Number Variants in the Kallikrein Gene Cluster

**DOI:** 10.1371/journal.pone.0069097

**Published:** 2013-07-22

**Authors:** Pernilla Lindahl, Torbjörn Säll, Anders Bjartell, Anna M. Johansson, Hans Lilja, Christer Halldén

**Affiliations:** 1 Department of Laboratory Medicine, Division of Clinical Chemistry, Lund University, Skåne University Hospital, Malmö, Sweden; 2 Biomedicine, Kristianstad University, Kristianstad, Sweden; 3 Department of Biology, Lund University, Lund, Sweden; 4 Department of Urology, Skåne University Hospital, Malmö, Sweden; 5 Department of Animal Breeding and Genetics, Swedish University of Agricultural Sciences, Uppsala, Sweden; 6 Departments of Laboratory Medicine, Surgery (Urology), and Medicine (Genitourinary Oncology), Memorial Sloan-Kettering Cancer Center, New York, New York, United States of America; 7 Nuffield Department of Surgical Sciences, University of Oxford, Oxford, United Kingdom; 8 Institute of Biomedical Technology, University of Tampere, Tampere, Finland; University of Patras, Greece

## Abstract

The kallikrein gene family (*KLK1*-*KLK15*) is the largest contiguous group of protease genes within the human genome and is associated with both risk and outcome of cancer and other diseases. We searched for copy number variants in all *KLK* genes using quantitative PCR analysis and analysis of inheritance patterns of single nucleotide polymorphisms. Two deletions were identified: one 2235-bp deletion in *KLK9* present in 1.2% of alleles, and one 3394-bp deletion in *KLK15* present in 4.0% of alleles. Each deletion eliminated one complete exon and created out-of-frame coding that eliminated the catalytic triad of the resulting truncated gene product, which therefore likely is a non-functional protein. Deletion breakpoints identified by DNA sequencing located the *KLK9* deletion breakpoint to a long interspersed element (LINE) repeated sequence, while the deletion in *KLK15* is located in a single copy sequence. To search for an association between each deletion and risk of prostate cancer (PC), we analyzed a cohort of 667 biopsied men (266 PC cases and 401 men with no evidence of PC at biopsy) using short deletion-specific PCR assays. There was no association between evidence of PC in this cohort and the presence of either gene deletion. Haplotyping revealed a single origin of each deletion, with most recent common ancestor estimates of 3000-8000 and 6000-14 000 years for the deletions in *KLK9* and *KLK15*, respectively. The presence of the deletions on the same haplotypes in 1000 Genomes data of both European and African populations indicate an early origin of both deletions. The old age in combination with homozygous presence of loss-of-function variants suggests that some kallikrein-related peptidases have non-essential functions.

## Introduction

Besides the extensively studied single nucleotide polymorphisms (SNPs) the human population harbors a large number of structural variants as well as short indels. Many of these appear to be neutral or nearly neutral whereas others have been associated with disease. Since this type of variation in general is more difficult to study than SNPs, much remains to be investigated regarding structural variants. The most easily detected structural variants are copy number variants (CNVs). The discovery and characterization of CNVs have largely been made using hybridization-based microarrays and next-generation sequencing [1]. A number of databases of CNVs have been established, including the Database of Genomic Variants (DGV; http://dgvbeta.tcag.ca/dgv/app/home?ref=NCBI36/hg18) which lists close to 300 000 CNVs that cover a large part of the genome. During the past 10 years, over 50 studies contributed information to this database and subsequent reports disseminated increasingly large data sets of CNVs. Many of these CNVs have been discovered in general surveys for variation, i.e. with no prior or primary selection for a specific phenotype. For example, Conrad et al. [2] investigated the occurrence of CNVs greater than ~500 bp in a subsample of the HapMap populations using microarrays with 42 million probes. They mapped 11 700 CNVs and generated reference CNV genotypes for approximately 5000 of these. During the last few years, there has been a tremendous increase in the number of reported CNVs as well as an increasing detail of their population frequencies. However, basic knowledge about their exact sizes and their breakpoint sequences are still missing at large. Conrad et al. [3] addressed this by characterizing more than 300 CNV breakpoints and identifying two major breakpoint signatures: 70% of the deletion breakpoints have 1-30 bp of microhomology, whereas 33% of deletion breakpoints contain 1-367 bp of inserted sequences. The co-occurrence of microhomology and inserted sequence was low, 10%. This suggests the presence of at least two major different mutational mechanisms. Exact definition of deletion breakpoints is critical for determining the precise functional impact of a CNV as well as allowing the construction of CNV-specific PCR assays aimed for identifying disease-associations.

Naturally, the effects of CNVs vary with their position, and there is growing interest in understanding the relationship between specific CNVs in the human genome and variation in different phenotypes. CNVs are involved in many genomic disorders, both monogenic diseases and complex disorders [4]. Disease-associated CNVs have been discovered when candidate genes for different diseases have been investigated, but a number of studies have also used information about known CNVs to investigate their association to disease. This strategy is exemplified by Craddock et al. [5] who investigated more than 3400 CNVs for their association with disease in 16 000 cases of 8 common diseases and 3000 shared controls. Although multiple associations were identified, common CNVs were concluded to be an unlikely source of substantial contributions to the genetic basis of common disease. Other studies have, however, identified a large number of associations with both rare and common CNVs in different diseases such as attention deficit hyperactivity disorder [6], Crohns disease [7] and schizophrenia [8].

Human kallikrein-related peptidase (*KLK*) genes are located on chromosome 19q13, 4 in a 270-kbp region and are the largest contiguous group of protease genes within the human genome. The *KLK* locus consists of 15 genes (*KLK1-15*), with lengths ranging from 4.4 to 11.0 kbp. With the exception of the primate-specific duplication of a predecessor gene into *KLK2* and *KLK3*, the *KLK* genes are transcribed from telomere to centromere. Still, all 15 *KLK* genes share the same exon/intron organization with 5 coding exons of equal lengths, and their proteins share a high degree of homology. They also share the same conserved translational start and stop sites, 10 conserved cysteine residues serving as template to enable correct folding, and the triad of catalytic codons [9]. *KLK*s are expressed in different tissues and have been implicated in a wide range of physiological processes. Multiple reports have suggested that *KLK* genes may be dysregulated in cancers. Several *KLK* genes have in previous studies shown significant associations with cancer risk and outcome, e.g. in breast cancer, ovarian cancer and prostate cancer [10,11]. Due to their early discovery, *KLK1*-*3* have been extensively studied and prostate-specific antigen (PSA), encoded by *KLK3*, is currently the most widely used biomarker for prostate cancer [12].

The present study comprehensively investigates the *KLK* gene cluster on 19q13.4 for the presence of CNVs. Two deletions were detected and carefully characterized in study cohorts of mainly European ancestry in terms of their population frequencies, number of origins, ages, breakpoint sequences and their association with prostate cancer.

## Material and Methods

### Ethics statement

This study uses 3 different materials to investigate the occurrence of CNVs in the kallikrein locus. The family material was obtained in a process including written informed consent from all participants. The second material was collected from volunteers in a process including verbal informed consent. These samples are completely anonymized. The third material was obtained in a process including written informed consent from all participants. These procedures have been approved by the Ethical Committee of Lund University and the Swedish Data Inspection Board.

### Study subjects

Three different study populations were investigated: the first is a family material, the second consists of anonymous unrelated individuals and the third consists of cases and controls for prostate cancer. To detect deletions based on the segregation of SNP markers in families, we used DNA isolated from whole blood from 190 individuals representing 40 three-generation families. A majority of these families were represented by two grandparents, two parents and one child (study population 1). To search for the presence of CNVs by quantitative PCR, DNA from 285 unrelated individuals from the general population were investigated. In cases where a low frequency (<5%) CNV was detected an additional 285 unrelated individuals were analyzed. The samples were collected at the Department of Laboratory Medicine, Skåne University Hospital (SUS) in Malmö and anonymized (study population 2). We also evaluated any association with prostate cancer in a cohort of men referred to the Department of Urology at Skåne University Hospital (SUS) Malmö during 2004-2010, due to either elevated levels of PSA in blood (≥3 ng/ml), suspicious digital rectal exam (DRE), or free-to-total PSA-ratio ≤20%. This cohort underwent prostate biopsy showing evidence of prostate cancer in 276 men and no evidence of prostate cancer in 412 men (study population 3). Clinical data for study population 3 is summarized in Table S1. Genomic DNA was extracted from blood collected in EDTA using QIAamp DNA Blood Maxi or Mini kits (Qiagen, Hilden, Germany), and DNA concentrations were determined by fluorometry using PicoGreen (Molecular Probes, Invitrogen, Eugene, OR, USA). Due to low DNA quantity a total of 10 cases and 11 controls were discarded from further analysis.

### SNP genotyping

The database dbSNP (www.ncbi.nlm.nih.gov/SNP) was searched for SNPs within the entire human *KLK* locus. A set of 97 SNP markers (Table S2) were selected for genotype analysis in 190 individuals from 40 three-generation families. All *KLK* genes were covered with tagSNPs (r^2^>0.8, MAF>0.05) and to these markers a limited number of previously investigated SNPs were added. In the screening for deletions using segregation of SNP markers, the necessary and sufficient criterion for deducing the presence of a deletion is that a parent-offspring pair is scored as ‘homozygous’ for different alleles. In reality they are then both heterozygous for the deletion and carry different alleles for the SNP investigated [13]. The subsequent characterization of the confirmed deletions used a subset of these SNP markers to analyze 285 unrelated individuals (Table S2). Genotypes were determined using the Sequenom MassARRAY MALDI-TOF system as previously described [14].

### PCR quantification

Relative copy numbers were determined using duplex TaqMan assays (Applied Biosystems, Foster City, CA, USA) and the CopyCaller Software (Applied Biosystems, v. 1.0). The duplex TaqMan PCR assays consisted of a FAM dye-labeled target assay and a VIC dye-labeled reference assay. The real-time PCR C_T_ data were used by the CopyCaller Software to calculate sample copy number values by relative quantification using the comparative C_T_ (∆∆C_T_) method. All genes were analyzed using at least one TaqMan assay. When a candidate CNV was found, a number of assays flanking the original assay were analyzed to confirm the finding and to roughly estimate the size of the CNV (Table S3). Four sample replicates were analyzed on an ABI Prism 7900HT Sequence Detection System (Applied Biosystems). The amplification was run using the following parameters: 95°C for 10 min, 40 cycles of 95°C for 15 s, and 60°C for 60 s. PCR reactions were performed in a total volume of 10 µl containing 10 ng template DNA, 5 µl TaqMan Genotyping Master Mix, 0.5 µl TaqMan Copy Number Assay, and 0.5 µl TaqMan Copy Number Reference Assay. The results were analyzed using the CopyCaller Software where the data was analyzed without a calibrator sample. Copy number predictions with less than 2 replicates and low quality data were eliminated from further study. Data of sufficient quality is defined as: 1) confidence values >95% and 2) Z-scores <2.69. The number of low-quality data points was calculated for 285 individuals and 15 assays. This was used to identify assays that did not work properly and samples with low DNA quality and quantity. A total of 7 individuals were eliminated and the remaining 278/285 individuals all produced high quality data for all assays.

### Microsatellite genotyping

A 400-kbp sequence containing the *KLK* locus was searched for tandem repeats using the Tandem Repeats Finder Software [15]. Repeat loci located in the vicinity of *KLK9* and *KLK15* that had more than 15 perfect repeat units were selected. Primers were designed using NCBI/Primer-BLAST (http://www.ncbi.nlm.nih.gov/tools/primer-blast/). Primer sequences are listed in Table S4. PCR reactions were performed in a total volume of 10 µl containing 20 ng of template DNA, 0.4 U Taq DNA polymerase (AmpliTaq GOLD, Applied Biosystems), 1X PCR buffer II, 2.5 mM MgCl_2_, 0.25 mM dNTPs, and 0.4 µM of each primer. Microsatellite markers were amplified using the following PCR conditions: 95°C for 12 min, 10 cycles of 94°C for 15 s, 60°C for 15 s, 72°C for 30 s, 20 cycles of 89°C for 15 s, 60°C for 15 s, 72°C for 30 s, and finally 72°C for 10 min. All reactions were run under the same conditions on a Veriti 96 Well Thermal Cycler (Applied Biosystems). PCR products were diluted in formamide and pooled before separation using capillary electrophoresis on an ABI Prism 310 Genetic Analyzer (Applied Biosystems). Data were analyzed using the GeneMarker Software (SoftGenetics, State College, PA, USA).

### Haplotyping and age estimation

Haplotypes of all deletion-carrying individuals were constructed using 68 SNP and 5 microsatellite markers distributed over the entire *KLK* locus. Two different methods were used to infer haplotypes in the deletion-carrying individuals. The first method involved the software PHASE v. 2.1 [16,17], using the deletions as dummy variables. The second was to manually infer the haplotypes. Since the data were obtained from single unrelated individuals, the haplotypes can be determined unambiguously only at homozygous loci. For the remaining loci, the haplotypes of the deletion-carrying individuals were constructed under the assumption that the deletion-carrying chromosomes also carried a common original haplotype that was consistently made as long as possible. Thus, only when two individuals were homozygous for different alleles were the haplotypes of the deletion-carrying chromosomes considered to be different. In every such case, the minor haplotype was considered to have experienced an exchange and the major haplotype was considered the original haplotype. This will be referred to below as the maximum principle and is explained in Text S1. The ages of the deletions were estimated using the software ESTIAGE [18]. This program uses the distribution of shared haplotype lengths and estimates the age to the most recent common ancestor (MRCA) of the sample, i.e. not to the origin of the mutation.

### Sequencing of deletion breakpoints

Long-range (LR) PCR systems encompassing the deletion breakpoints were designed using NCBI/Primer-BLAST (Table S5). PCR was performed in a total reaction volume of 10 µl, containing 20 ng of template DNA, 0.4 U LongRange PCR enzyme mix (Qiagen), 1X LongRange PCR buffer, 0.2 mM dNTPs and 0.4 µM of each primer. PCR was performed using the following PCR conditions: 93°C for 3 min, 6 cycles at 93°C for 30 s, 65°C for 30 s (-1°C every cycle), 68°C for 1 min/kbp, followed by 30 cycles at 93°C for 30 s, incubation at annealing temperature for 30 s, 68°C for 1 min/kbp, followed by a final extension step at 68°C for 10 min. The annealing temperatures were 58°C and 62°C for the *KLK9* and *KLK15* systems, respectively. The PCR products were visualized on 1% agarose gels and analyzed with the Image Lab Software version 3.0 (Bio-Rad Laboratories). To determine the DNA sequence of the LR-PCR amplicons a number of sequencing PCR primers were designed to cover the region with the deletion breakpoint (Table S5). PCR products were treated with ExoSAP-IT (USB Corporation, Cleveland, OH, USA) following the manufacturer’s instructions. DNA sequencing was performed in a total volume of 20 µl containing 10 µl of the LR-PCR product (~200 ng DNA), 0.5X Big Dye sequencing ready reaction premix (Big Dye Terminator v. 1.1 kit, Applied Biosystems), 0.5X Big Dye Sequencing Buffer, and 3.2 pmol primer. The following PCR conditions were used: 96°C for 1 min, 25 cycles of 96°C for 10 s, 50°C for 5 s and 60°C for 4 min. The sequencing reactions were purified with DyeEx 2.0 spin kit (Qiagen) according to the manufacturer’s instructions and analyzed on an ABI Prism 310 Genetic Analyzer (Applied Biosystems). Sequencing data were subsequently aligned and analyzed using the SeqScape Software (Applied Biosystems). All sequence data generated in this study have been submitted to the GenBank database (accession numbers KF053147 and KF053148).

### Deletion-specific PCR assays

Deletion-specific PCR systems were designed using the information about the deletion breakpoint sequences in *KLK9* and *KLK15* (Table S5). PCR was performed in a total reaction volume of 10 µl, containing 20 ng of template DNA, 0.25 U KAPATaq Extra HotStart DNA Polymerase (Kapa Biosystems, Cape Town, South Africa), 1X KAPATaq Extra Buffer, 1.75 mM MgCl_2_, 0.3 mM dNTPs, and 0.4 µM of each primer. The following PCR conditions were used: 94°C for 3 min, 35 cycles at 94°C for 15 s, 66°C for 15 s, 72°C for 1 min/kbp, followed by a final extension step at 72°C for 10 min. Optimization of each PCR system was performed using individuals known to have deletions in the *KLK9* and *KLK15* genes as well as wild type individuals. Screening for deletions was performed on a Veriti 384 Well Thermal Cycler (Applied Biosystems).

## Results

### Detection of deletions using segregation of markers in pedigrees

**Figure 1:**
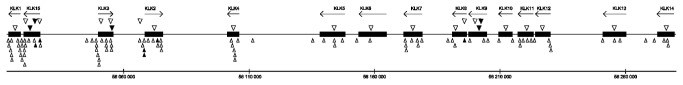
Map of the kallikrein gene locus showing positions of genes, copy number- and SNP assays. Chromosome positions are according to the human reference sequence of NCBI, Build 36.3. Copy number (CN)-assays are indicated by triangles above the line and SNP assays are indicated by triangles below the line. CN-assays next to the line denote assays in the first screen, whereas assays in the second row above the line were used for confirmation. Filled symbols indicate an assay result compatible with the presence of a deletion.

To screen for deletions in the *KLK* gene cluster a total of 97 SNPs covering all 15 *KLK* genes (Figure 1) were analyzed using DNA from 190 individuals in 40 three-generation families. Genotype patterns indicating the presence of null-alleles, i.e. cases where parent-offspring pairs were homozygous for different alleles, were identified for 6 SNP markers in a total of 13 families. In the *KLK15* gene, we found one null-allele for the SNP marker rs266114 in 6/40 families analyzed, and identified an additional null-allele in a flanking SNP marker (rs266115) in one of the 6 families. Further, null-alleles in the *KLK2* gene were identified for 3 SNP markers (809prom, rs11670728 and rs198972). By using the rs11670728 marker, we identified null-alleles in 2 families, whereas the other 2 markers demonstrated presence of null-alleles in one unique family each. Finally, the SNP marker rs1701947 identified null-alleles in the *KLK8* gene in 3/40 families.

The observed null-alleles can either be due to mutations in primer annealing sequences or due to the presence of deletions. The observation of null-alleles for several consecutive SNP markers in the same family strongly increases the likelihood that a deletion is present. Since the 2 *KLK15* markers showing null-alleles had non-overlapping primer sequences and their null-alleles were located in close proximity to each other on the same chromosome, these null-alleles are very likely due to a deletion. In *KLK2* and *KLK8*, the consecutive flanking markers do not support the presence of deletions. Therefore, we believe that these null-alleles are more likely to result from mutations occurring in primer annealing sites.

Testing for Hardy-Weinberg equilibrium identified 5 significant SNPs. Four of these were associated with the null-alleles demonstrated above. Two were directly involved in null-alleles, rs266115 in *KLK15* and rs1701947 in *KLK8* and the remaining 2 (rs1722561 and rs2659096) were directly flanking rs1701947. The fifth resides in the *KLK3* gene (rs266882). In all 5 cases there were more homozygous individuals than anticipated, which is compatible with the existence of deletions.

### Detection of CNVs using quantitative PCR

The presence of CNVs in DNA isolated from 278/285 unrelated individuals was analyzed using quantitative PCR. Relative copy numbers were determined using one assay for each of the 15 *KLK* genes. Candidate deletions were identified in *KLK15*, *KLK3*, and *KLK9* whereas no candidate duplications were detected (Figure 1). Candidate deletions in *KLK15* were identified in a total of 20/278 (7.2%) individuals investigated, two of whom were homozygotes. Due to the detection of candidate deletions in *KLK3* and *KLK9* in 1 and 4 individuals, respectively, we also analyzed DNA isolated from an additional set of 285 unrelated individuals. In the combined data set of 563 individuals only a single individual showed a candidate deletion in *KLK3*, whereas 13/563 (2.3%) individuals carried a candidate deletion in *KLK9*, with 1 out of 13 being homozygous for the tentative deletion in *KLK9*.

To confirm the candidate deletions, additional assays flanking the positions of the original SNP and TaqMan assays were analyzed using DNA from the deletions-carrying individuals and a subset of controls. In *KLK15*, two consecutive assays confirmed the deletions and their zygozity in all 20 individuals discussed above. The estimate of the corresponding deletion frequency is 0.040 (95% confidence interval [CI] 0.023, 0.056). In *KLK9* two consecutive TaqMan assays confirmed the presence of a deletion in the 13 individuals; 12 heterozygotes and 1 homozygote. This corresponds to a deletion frequency of 0.012 (95% CI 0.006, 0.019). In *KLK3* two additional TaqMan assays failed to confirm a deletion in the single individual with a candidate deletion. Also in *KLK2* and *KLK8* additional assays failed to detect CNVs, which suggests mutations in primer annealing sequences rather than deletions in all of the cases where we initially suspected a tentative deletion in *KLK2*, *KLK3*, or *KLK8*.

### Characterization of deletions in *KLK9* and *KLK15* by haplotyping

A total of 68 SNP and 5 microsatellite markers distributed over the entire *KLK* locus were used to analyze all 33 deletion-carrying individuals and 62 randomly selected deletion-negative individuals to determine whether the deletions are identical by descent or recurrent mutations.

The results of the quantitative PCR showed that one of the 13 individuals carrying the *KLK9* deletion was homozygous for the deletion. Six of the SNP markers flanking the deletion were also homozygous in this individual, thus defining an unambiguous ‘deletion haplotype’ (Table 1, sample 469). The 6 SNP alleles defining the deletion haplotype were also present in all 12 individuals heterozygous for the deletion. It is thus possible that the deletion-carrying chromosomes in the heterozygotes carry the same deletion haplotype observed in the homozygote. The complementary haplotypes could be constructed in the deletion heterozygotes assuming the presence of the deletion haplotype. These complementary haplotypes had frequencies as expected from the known haplotype frequencies of the background population (Table 1). This supports the assumption of a common deletion haplotype. The deletion haplotype had a relatively low frequency, both among the individuals from the HapMap CEU population (3%) and among the 62 deletion-negative individuals (7%). Analyzing microsatellite data, the two flanking alleles 208 and 147 were present on 13 out of 14 deletion chromosomes. Among the deletion-negative individuals the deletion haplotype occurred at an estimated frequency of 6% (Table S6).

**Table 1:** Haplotypes estimated in individuals carrying deletions in the *KLK9* gene.

**SampleID**	**rs1612902**	**Deletion^*a*^**	**rs952109**	**rs885756**	**rs10426**	**rs1048344**	**rs3745539**	**HapMapfrequency^*b*^**
15	**G**	**D**	**T**	**T**	**T**	**A**	**C**	0.03
	G	W	T	T	C	A	C	0.16
110	**G**	**D**	**T**	**T**	**T**	**A**	**C**	0.03
	A	W	C	T	C	A	C	0.30
224	**G**	**D**	**T**	**T**	**T**	**A**	**C**	0.03
	G	W	T	T	C	A	T	0.04
285	**G**	**D**	**T**	**T**	**T**	**A**	**C**	0.03
	A	W	C	T	C	G	C	0.03
292	**G**	**D**	**T**	**T**	**T**	**A**	**C**	0.03
	A	W	C	T	T	A	C	0.18
316	**G**	**D**	**T**	**T**	**T**	**A**	**C**	0.03
	A	W	C	T	C	A	C	0.30
320	**G**	**D**	**T**	**T**	**T**	**A**	**C**	0.03
	G	W	T	T	C	G	C	0.18
328	**G**	**D**	**T**	**T**	**T**	**A**	**C**	0.03
	A	W	C	C	C	A	C	0.03
394	**G**	**D**	**T**	**T**	**T**	**A**	**C**	0.03
	G	W	T	T	C	A	C	0.16
437	**G**	**D**	**T**	**T**	**T**	**A**	**C**	0.03
	G	W	T	T	C	A	T	0.04
465	**G**	**D**	**T**	**T**	**T**	**A**	**C**	0.03
	A	W	T	T	C	A	T	0.00
469	**G**	**D**	**T**	**T**	**T**	**A**	**C**	0.03
	**G**	**D**	**T**	**T**	**T**	**A**	**C**	0.03
513	**G**	**D**	**T**	**T**	**T**	**A**	**C**	0.03
	G	W	T	T	C	A	C	0.16

a D, deletion; W, wild type.

b The deletion haplotypes and the complementary haplotypes were constructed as given in Materials and methods. HapMap frequencies are given for each constructed haplotype for the European (CEU) population.

Deletion haplotypes in bold.

The haploblock structure of the *KLK* gene region was subsequently determined using Haploview v. 4.1 [19] on HapMap data from the CEU population. The *KLK9* deletion is located in a 28-kbp haploblock containing the same 6 SNPs used to define the deletion haplotype (Figure S1). The presumed single origin of the *KLK9* deletion prompted us to estimate its age. The first step was to haplotype all individuals for the remaining SNPs (i.e. the SNPs that were not homozygous in the individual that was homozygous for the deletion). This was done using either PHASE or the maximum principle strategy described in Materials and methods and Text S1. When PHASE was used, the deletion was included in the data and the haplotypes for the 6 SNPs described above were fixed. The two methods resulted in two sets of possible haplotype configurations for the 13 deletion-carrying individuals, where the maximum principle gave considerably longer common haplotypes among the individuals (Table S7). Both datasets were then analyzed using ESTIAGE. When PHASE was used to construct haplotypes, the estimate of the age of the deletion haplotype was 258 generations (95% CI 70, 394), whereas the maximum principle resulted in an estimate of 95 generations (95% CI 57, 158).

A total of 20 individuals were found to carry the deletion in *KLK15* (Table 2). Two were homozygous for the deletion and homozygous for an identical haplotype containing 12 SNPs (Table S8). It was thus possible to construct haplotypes in the same way as was done for *KLK9*. When the 18 deletion heterozygotes were investigated, they all carried the alleles of the haplotype found in the two homozygotes. Thus, the data for the *KLK15* deletion are also completely compatible with the presence of a single deletion haplotype. In this case the deletion haplotype is even less frequent than in the case of *KLK9*. This haplotype cannot be found in the HapMap CEU population and was observed in a single chromosome in the 62 deletion-negative controls, i.e. a frequency of 0.008. The *KLK15* deletion is located in an 8-kbp haploblock (Figure S1). Constructing haplotypes according to the model described above yielded age estimates of 476 (320,711) and 190 (126,288) generations for the PHASE and maximum principle strategies, respectively (Table S8).

**Table 2:** Haplotypes estimated in individuals carrying deletions in the *KLK15* gene.

**SampleID**	**rs2659058**	**rs3212820**	**rs5514**	**rs3212815**	**rs3212855**	**rs3212810**	**Deletion^*a*^**	**rs3745523**	**HapMapfrequency^*b*^**
31	**A**	**G**	**T**	**T**	**A**	**G**	**D**	**C**	0.00
	A	G	G	C	A	G	W	C	0.22
38	**A**	**G**	**T**	**T**	**A**	**G**	**D**	**C**	0.00
	G	G	G	C	A	G	W	C	0.29
56	**A**	**G**	**T**	**T**	**A**	**G**	**D**	**C**	0.00
	**A**	**G**	**T**	**T**	**A**	**G**	**D**	**C**	0.00
83	**A**	**G**	**T**	**T**	**A**	**G**	**D**	**C**	0.00
	A	T	G	C	A	A	W	C	0.18
91	**A**	**G**	**T**	**T**	**A**	**G**	**D**	**C**	0.00
	A	G	G	C	C	G	W	C	0.06
112	**A**	**G**	**T**	**T**	**A**	**G**	**D**	**C**	0.00
	G	G	G	C	A	G	W	T	0.07
122	**A**	**G**	**T**	**T**	**A**	**G**	**D**	**C**	0.00
	A	G	G	C	C	G	W	C	0.06
142	**A**	**G**	**T**	**T**	**A**	**G**	**D**	**C**	0.00
	A	G	G	C	C	G	W	C	0.06
144	**A**	**G**	**T**	**T**	**A**	**G**	**D**	**C**	0.00
	G	G	G	C	A	G	W	C	0.29
152	**A**	**G**	**T**	**T**	**A**	**G**	**D**	**C**	0.00
	G	G	G	C	A	G	W	C	0.29
161	**A**	**G**	**T**	**T**	**A**	**G**	**D**	**C**	0.00
	G	G	G	C	A	G	W	C	0.29
174	**A**	**G**	**T**	**T**	**A**	**G**	**D**	**C**	0.00
	A	T	G	C	A	A	W	T	0.04
188	**A**	**G**	**T**	**T**	**A**	**G**	**D**	**C**	0.00
	**A**	**G**	**T**	**T**	**A**	**G**	**D**	**C**	0.00
208	**A**	**G**	**T**	**T**	**A**	**G**	**D**	**C**	0.00
	A	G	G	C	C	G	W	T	0.03
221	**A**	**G**	**T**	**T**	**A**	**G**	**D**	**C**	0.00
	A	G	G	C	A	G	W	T	0.11
227	**A**	**G**	**T**	**T**	**A**	**G**	**D**	**C**	0.00
	A	G	G	C	A	G	W	T	0.11
239	**A**	**G**	**T**	**T**	**A**	**G**	**D**	**C**	0.00
	A	G	G	C	C	G	W	T	0.03
258	**A**	**G**	**T**	**T**	**A**	**G**	**D**	**C**	0.00
	A	T	G	C	A	A	W	C	0.18
266	**A**	**G**	**T**	**T**	**A**	**G**	**D**	**C**	0.00
	G	G	G	C	A	G	W	C	0.29
279	**A**	**G**	**T**	**T**	**A**	**G**	**D**	**C**	0.00
	G	G	G	C	A	G	W	C	0.29

a D, deletion; W, wild type.

b The deletion haplotypes and the complementary haplotypes were constructed as given in Materials and methods. HapMap frequencies are given for each constructed haplotype for the European (CEU) population. Deletion haplotypes in bold.

### Characterization of deletion breakpoints in *KLK9* and *KLK15*

**Figure 2:**
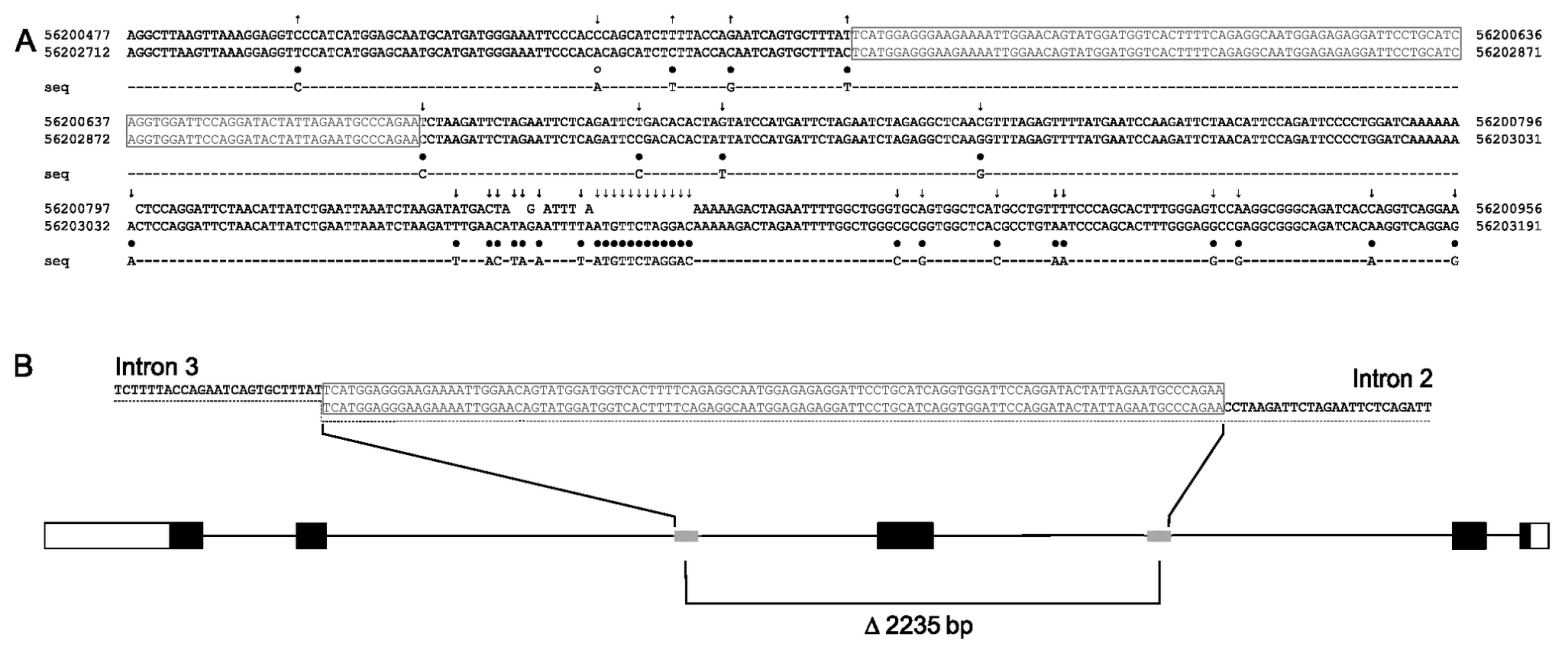
Location of the breakpoint sequences of the deletion in the *KLK9* gene. (A) An alignment of two repeated sequences of the *KLK9* gene is shown together with their chromosome positions (Build 36.3). Dots highlight positions where the two sequences are different (unfilled dot shows a polymorphic nucleotide position). The line marked ‘seq’ is the result of the DNA sequencing of deletion-carrying individuals. Arrows indicate which of the homologous sequences agrees with the sequence from the deletion-carrying individuals: an arrow pointing up shows that the upper sequence agrees and an arrow pointing down shows that the lower sequence agrees. The differences between the two homologous sequences, indicated by boxes, limit the exact breakpoint position. (B) Breakpoints of the deletion are located between nucleotides 56 200 564-56 200 671 in intron 3 and nucleotides 56 202 799-56 202 906 in intron 2 of the *KLK9* gene, which results in a 2235-bp deletion eliminating exon 3.

**Figure 3:**
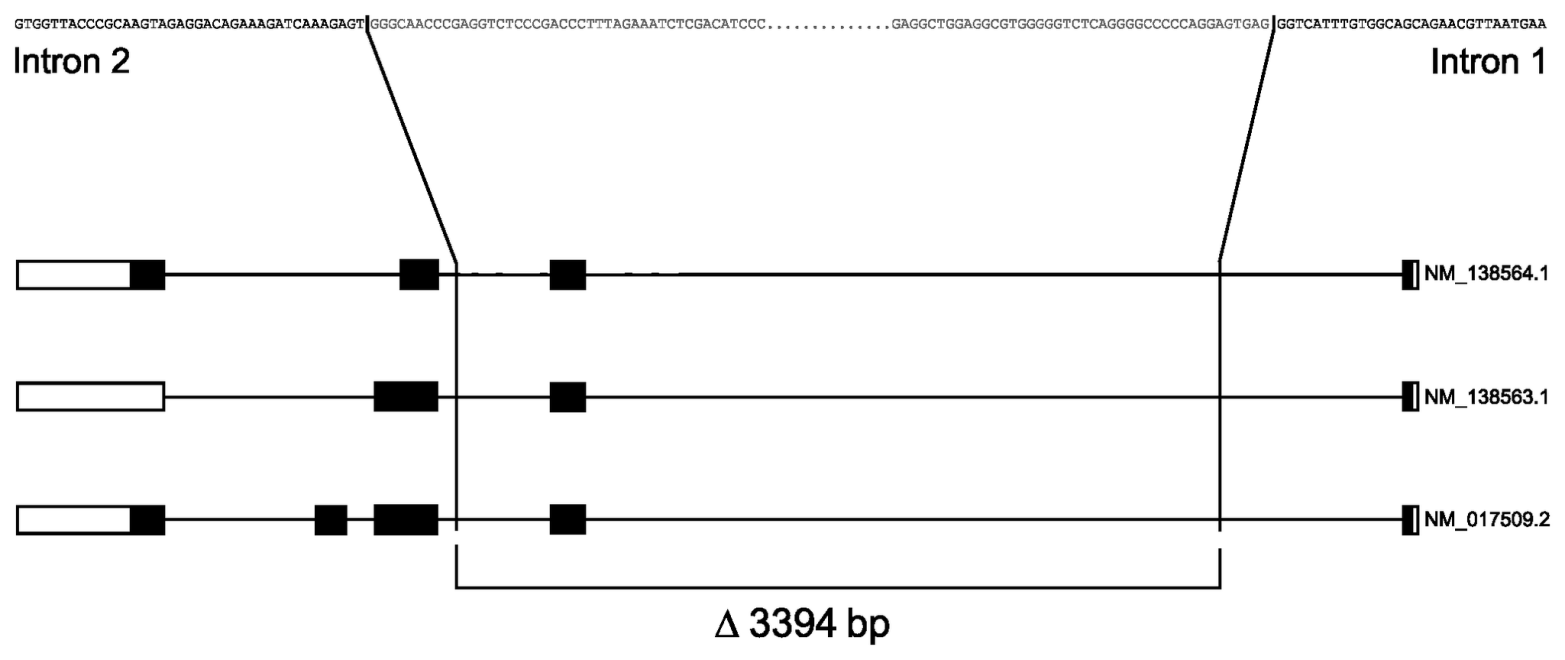
Location of the breakpoint sequences of the deletion in the *KLK15* gene. The deletion in the *KLK15* gene is located between nucleotide 56 022 311 in intron 2 and nucleotide 56 025 704 in intron 1 of the *KLK15* gene, which results in a 3394-bp deletion eliminating exon 2.

The chromosomal regions covering the deletion breakpoints were subsequently resequenced to further characterize these deletions. The deletion breakpoints in *KLK9* are located in an interspersed repeated sequence (L3/CR1). The two homologous sequences were aligned and variations between them were identified (Figure 2A). The data show that the centromeric breakpoint of the deletion in *KLK9* is located between nucleotide positions 56 200 564 and 56 200 671, and the telomeric breakpoint is located between nucleotide positions 56 202 799 and 56 202 906, resulting in a 2235-bp deletion including the complete exon 3 in *KLK9* (Figure 2B). The deletion in *KLK15* covers chromosome positions 56 022 311 to 56 025 704, corresponding to a 3394-bp deletion including the complete exon 2 in *KLK15* (Figure 3). Both deletions eliminate a complete exon and create in both cases out-of-frame coding, thereby eliminating amino acids that are part of the catalytic sites of their respective proteins. In *KLK9* the combined effect of missing codons and frame-shift reduces the protein from 250 amino acids to 113 where only 68 are identical to the original protein. *KLK15* exists as three different transcripts (161, 171 and 256 amino acids). In all cases the deletion reduces the protein to 16 amino acids. Thus, the resulting truncated proteins are most likely non-functional.

### Screening for the *KLK9* and *KLK15* deletions in prostate cancer patients

To search for an association between the presence or absence of the deletions in *KLK9* and *KLK15* and prostate cancer (PC), DNA isolated from 266 men with evidence of PC at prostate biopsy and 401 men with no evidence of PC at biopsy (see Table S1 for a summary of clinical data) were analyzed using short deletion-specific PCR assays. To confirm the deletions and determine their zygosity we also analyzed these individuals using TaqMan assays. The *KLK9* deletion was detected in a total of 22 heterozygous individuals. The estimated frequency of the deletion among the 266 PC cases was 0.017 (9/532), with the same frequency among men with no evidence of PC at biopsy, 0.016 (13/802). We identified the *KLK15* deletion in a total of 45 heterozygous individuals. The estimated allele frequency of the deletion in the PC cases was 0.041 (22/532), compared to 0.029 (23/802) among the men with benign histology at prostate biopsy. When tested by a chi-square homogeneity test none of these deletion-frequencies are significantly different (χ^2^=0.37; df = 1; *P*=0.54) for the *KLK9* deletion and (χ^2^=3.2; df = 1; *P*=0.08) for the *KLK15* deletion respectively. The *KLK9* deletion had a frequency of 0.012 in the general population sample whereas in the referral cohort of men who underwent prostate biopsy the frequency was 0.016. The corresponding frequencies for the *KLK15* deletion were 0.040 and 0.033. These two populations have similar frequencies of both deletions, thus in effect serving as a replication of the first frequency observations.

## Discussion

A comprehensive search for CNVs in the *KLK* gene family revealed two deletions, one in *KLK9* and one in *KLK15*. Both deletions are located on single rare haplotypes indicating single origins. Both deletions eliminate an entire exon, resulting in truncated proteins. Despite this, both deletions reach frequencies which define them as polymorphisms (>1%), the *KLK9* deletion with an allele frequency of 1.2% and the *KLK15* deletion with an allele frequency of 4.0%. In addition, both deletions are present in homozygous form at frequencies roughly as expected from their allele frequencies, indicating only a limited deleterious effect even in homozygous form (Table 3). The observation that large deletions reach frequencies above 1% could, in principle, be caused by positive selection for the deletion. However, since *KLK9* and *KLK15* have persisted as active genes in humans, and since homologues also exist in the chimpanzee, a positive selection for null-alleles over a long time period can be excluded. Extensive resequencing of all *KLK* genes [14] revealed no marked accumulation of mutations in *KLK9* and *KLK15* compared with the other *KLK* genes, arguing against selection also at a shorter time scale.

**Table 3:** Summary table of the deletions in *KLK9* and *KLK15*.

		***KLK9***	***KLK15***
**Chromosome position^*a*^**	Start	56 200 564-56 200 671	56 022 311
	Stop	56 202 799-56 202 906	56 025 704
**Deletion size (bp)**		2235	3394
**Allele frequency^*b*^**		0.012 (0.006, 0.019)	0.040 (0.023, 0.056)
**Homozygotes**		1/563	2/278
**Single origin**		Yes	Yes
**Age estimation^*b*,*c*^**	PHASE	258 (170, 394)	476 (320, 711)
	Maximum principle	95 (57, 158)	190 (126, 288)

a NCBI Build 36.3, chromosome 19.

b 95% confidence intervals within paranthesis.

c Generations.

When testing for association with PC no effect could be demonstrated. However, the power is limited due to the low frequencies of the deletion variants; a simulation shows that in the case of the *KLK15* deletion the power is 0.26 for an odds ratio of 1.4. Still, the results clearly show that if effects exist they are limited. In addition, the sample used in the association test provides an independent population sample to the initial screening population, allowing a replication of the frequency estimates of the two deletions. Given that *KLK9* and *KLK15* are weakly expressed in most tissues including the prostate [20] it is not surprising that no copy number association with prostate cancer was found.

The times to the MRCA were determined from two different estimates of the lengths of the common haplotypes in the chromosomes carrying the deletions. There are thus two size estimates per deletion and these estimates vary considerably. However, there are good reasons to believe that the correct size is within our estimates. Some of the haplotype configurations constructed by PHASE lead to overestimates of the times to the MRCA. On the other hand, the haplotypes constructed from the maximum principle are the longest possible for this data set and therefore most likely lead to underestimates of the ages. Using a generation time of 30 years, MRCA time estimates in the size ranges 3000-8000 years for *KLK9* and 6000-14 000 years for *KLK15* were obtained. This indicates that these deletions are likely to be found in other European populations.

To screen for the occurrence of the deletions in other populations, the CEU (European) and YRI (African) populations in the 1000 Genomes data set were investigated. A *KLK9* deletion mapping to the same locus and of exactly the same length as in the present study was found in both populations. In the CEU population the allele frequency was 0.022 and in the YRI population it was 0.006. A *KLK15* deletion of the same length and position as reported here occurred in a frequency of 0.033 in the CEU population and a frequency of 0.014 in the YRI population. Thus, as suggested from the age estimates both deletions are spread in the European population. In addition, both deletions were detected also in the African population compatible with an early origin in human history of these deletions. Haplotyping of the CEU and YRI deletion chromosomes revealed the presence of the same *KLK9* and *KLK15* deletion haplotypes also in these populations. Given the allele frequency of a mutation, its age can also be estimated under the assumption of neutrality using the method of Kimura and Otha [21]. This method provides a means to calculate the overall expected age of a neutral allele. These calculations require an estimate of the long-term effective population size of the studied population. When all humanity is concerned, it is common to use 10 000 individuals as the effective population size. In the present case, the sample is taken from a limited section of humanity, and we have therefore used 5000 individuals, the same value used by Reish et al. [22]. Using the Kimura and Otha [21] expressions, the estimate of the overall expected age for the deletion in *KLK9* was 1100 generations (33 000 years). For *KLK15* the corresponding age estimate was 2682 generations (80 460 years). These deletion age estimates are fully compatible with the presence of the deletions both in the European and African populations. Taken together, the identical deletion haplotypes and the deletion age estimates strongly indicate that the origins of both deletions predate the out of Africa migration.

With the exception of *KLK8*, *KLK10*, *KLK11* and *KLK12*, all *KLK* genes have been reported to contain CNVs in the DGV. The reported CNVs partly overlap and vary both in size and frequency. Many have been detected in single individuals only. Deletions found in *KLK9* and *KLK15* have been observed in prior studies, but have not been fully characterized. For example, in a cohort of 2026 individuals, Shaikh et al. [23] identified two deletions in the *KLK15* gene, one with an estimated size of 1739 bp in 22 individuals and one 5408-bp deletion in 2 individuals. In addition, a 2307-bp deletion in the *KLK15* gene was detected in 38 out of 1184 individuals [24]. All three deletions were observed in heterozygous form and are probably identical with the *KLK15* deletion reported in the present study. The varying size estimates could be due to the limited resolution of SNP chip data. Conrad et al. [2] observed a 1663-bp deletion in the *KLK9* gene in 4 out of 450 individuals from the HapMap project. The position given for this deletion overlaps the position of the *KLK9* deletion reported in the present study, and they probably represent the same deletion.

MacArthur et al. [25] made an extensive search for loss of function (LoF) variants including CNVs in 185 subjects from the 1000 Genome project. After careful filtering they found that an average human carries approximately 100 LoF variants with 20-25 of these in homozygous form. These individuals are reported as phenotypically normal. They furthermore searched for common features of LoF tolerant genes and found that members of gene families, in particular those with highly homologous family members, are more likely to tolerate LoF mutations. In addition, MacArthur and coworkers [25] found mutations in several members of the *KLK* gene family, including *KLK15*. Thus, our observation concerning the deletions in *KLK9* and *KLK15* are in line with their findings.

## Supporting Information

Text S1 Description of the maximum principle haplotyping method. (DOC)

Figure S1 Pattern of linkage disequilibrium (LD) for the HapMap CEU population. SNPs used to characterize the deletions in *KLK15* and *KLK9* with a frequency in the HapMap CEU population were used. LD is estimated and given as D'. Low LOD (<2) and low D' (<1) are shown in white, low LOD and perfect D' (1) are shown in blue, high LOD (#2) and low D' are shown in shades of pink/red and high LOD and perfect D' are shown in bright red. Positions of SNPs are given relative to the deletions in *KLK15* and *KLK9*. The filled arrows indicate the positions of the deleted regions, the deleted region in *KLK15* in-between marker rs3212810 and rs3745523 and the deleted region in *KLK9* in-between markers rs1612902 and rs952109.
(TIF)

Table S1 Patient characteristics by biopsy outcome. All values are median (IQR) or frequency (percent).
(XLS)

Table S2 SNP markers in the kallikrein gene locus analyzed in this study. (A) SNPs used in the first screening for deletions in the kallikrein locus. (B) SNPs used in haplotyping of the identified deletions.
(XLS)

Table S3 Copy number assays in the kallikrein gene locus analyzed in this study. The assay information is from the Applied Biosystems homepage (http://www.appliedbiosystems.com/absite/us/en/home.html). (A) Assays used in the first screening for CNVs. We used one assay per gene. (B) Assays used to confirm the findings from the SNP study and the first part of the CNV study.
(XLS)

Table S4 Microsatellite markers analyzed in the kallikrein gene locus. (XLS)

Table S5 Primer systems used in (A) long-range PCR, (B) sequencing of the deletion breakpoints and (C) deletion specific assays. (XLS)

Table S6 Alleles at microsatellite loci in (A) controls, (B) individuals with deletion in *KLK9* and (C) individuals with deletion in *KLK15*. Individuals homozygous for the deletion are marked grey. (XLS)

Table S7 Deletion haplotypes estimated using (A) PHASE and (B) the maximum principle for all individuals carrying deletions in the *KLK9* gene. (XLS)

Table S8 Deletion haplotypes estimated using (A) PHASE and (B) the maximum principle for all individuals carrying deletions in the *KLK15* gene. (XLS)
