# Supplementary material for: Copy Number Variants in the Kallikrein Gene Cluster
Source: PLoS One. 2013 Jul 22;8(7):e69097. doi: 10.1371/journal.pone.0069097 (PMC3718828; doi:10.1371/journal.pone.0069097)
Supplement: Text S1 — (DOC) [file pone.0069097.s001.doc]

**Text S1.** Description of the maximum principle haplotyping method.

The principle aims at consistently creating as long common haplotypes as possible. Consider the four uppermost individuals in Table S7B, i.e. ‘15’, ‘110’, ‘224’ and ‘285’. Starting from the deletion, the first 10 SNPs to the left of the deletion are homozygous. At rs268909 individual ‘110’ differs and since only this individual is different it is assumed that it carries a recombinant chromosome and that the others carry the original haplotype. It is also seen that ‘285’ has missing data for this marker. Since the principle aims at consistently creating as long common haplotypes as possible ‘285’ is assumed to carry the founder haplotype at this SNP. Using this principle the shared haplotypes among the deletion carriers are made as long as possible given the available data.
