# Supplementary figures and images for: Copy Number Variants in the Kallikrein Gene Cluster
Source: PLoS One. 2013 Jul 22;8(7):e69097. doi: 10.1371/journal.pone.0069097 (PMC3718828; doi:10.1371/journal.pone.0069097)

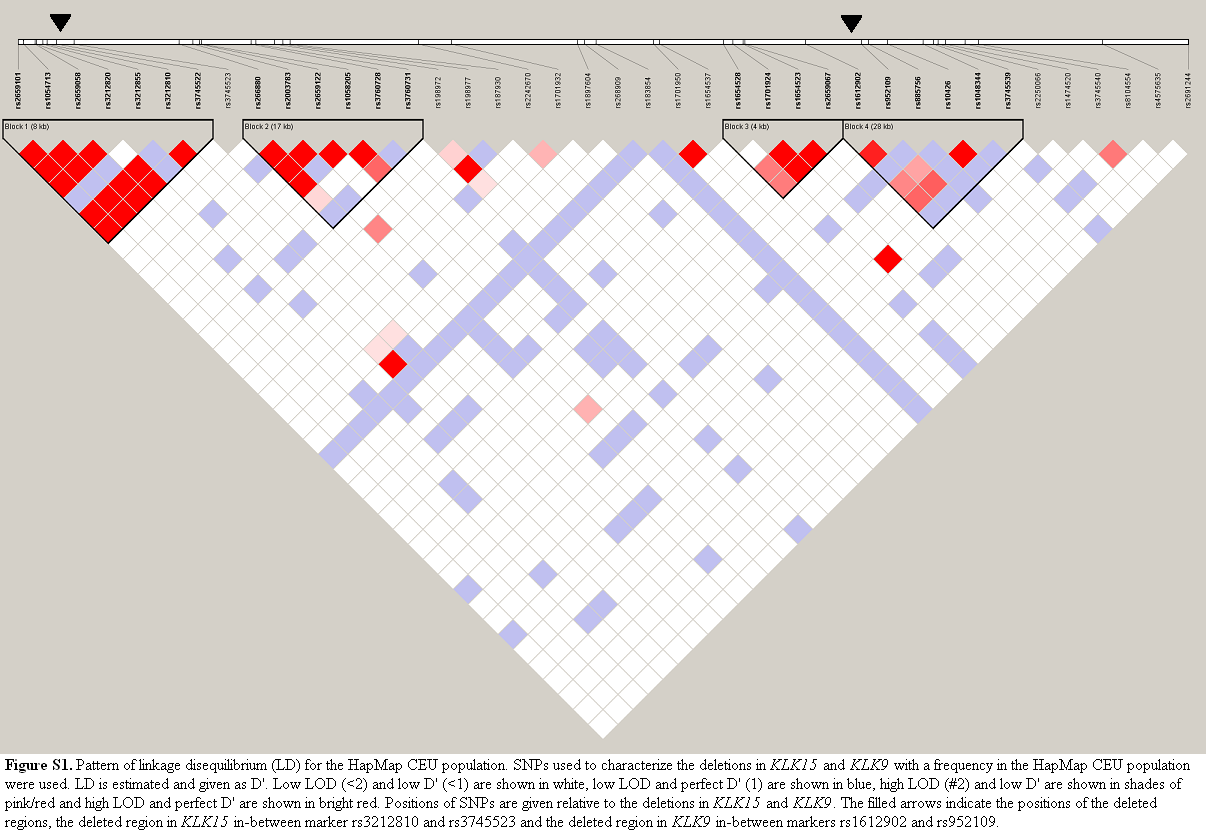

Supplement: Figure S1 — LD is estimated and given as D'. Low LOD (<2) and low D' (<1) are shown in white, low LOD and perfect D' (1) are shown in blue, high LOD (#2) and low D' are shown in shades of pink/red and high LOD and perfect D' are shown in bright red. Positions of SNPs are given relative to the deletions in KLK15 and KLK9. The filled arrows indicate the positions of the deleted regions, the deleted region in KLK15 in-between marker rs3212810 and rs3745523 and the deleted region in KLK9 in-between markers rs1612902 and rs952109. (TIF) [file pone.0069097.s002.tif]
